# Co-expression of adjacent genes in yeast cannot be simply attributed to shared regulatory system

**DOI:** 10.1186/1471-2164-8-352

**Published:** 2007-10-03

**Authors:** Huai-Kuang Tsai, Cindy PC Su, Mei-Yeh J Lu, Ching-Hua Shih, Daryi Wang

**Affiliations:** 1 Institute of Information Science, Academia Sinica, Taipei 115, Taiwan; 2 Research Center for Information Technology Innovation, Academia Sinica, Taipei 115, Taiwan; 3 Research Center for Biodiversity, Academia Sinica, Taipei 115, Taiwan; 4 Genomics Research Center, Academia Sinica, Taipei 115, Taiwan; 5 Department of Ecology and Evolutionary Biology, Rice University, Houston, TX 77005, USA

## Abstract

**Background:**

Adjacent gene pairs in the yeast genome have a tendency to express concurrently. Sharing of regulatory elements within the intergenic region of those adjacent gene pairs was often considered the major mechanism responsible for such co-expression. However, it is still in debate to what extent that common transcription factors (TFs) contribute to the co-expression of adjacent genes. In order to resolve the evolutionary aspect of this issue, we investigated the conservation of adjacent pairs in five yeast species. By using the information for TF binding sites in promoter regions available from the MYBS database , the ratios of TF-sharing pairs among all the adjacent pairs in yeast genomes were analyzed. The levels of co-expression in different adjacent patterns were also compared.

**Results:**

Our analyses showed that the proportion of adjacent pairs conserved in five yeast species is relatively low compared to that in the mammalian lineage. The proportion was also low for adjacent gene pairs with shared TFs. Particularly, the statistical analysis suggested that co-expression of adjacent gene pairs was not noticeably associated with the sharing of TFs in these pairs. We further proposed a case of the PAC (polymerase A and C) and RRPE (rRNA processing element) motifs which co-regulate divergent/bidirectional pairs, and found that the shared TFs were not significantly relevant to co-expression of divergent promoters among adjacent genes.

**Conclusion:**

Our findings suggested that the commonly shared *cis*-regulatory system does not solely contribute to the co-expression of adjacent gene pairs in yeast genome. Therefore we believe that during evolution yeasts have developed a sophisticated regulatory system that integrates both TF-based and non-TF based mechanisms(s) for concurrent regulation of neighboring genes in response to various environmental changes.

## Background

The arrangement and orientation of genes in genomes is often shaped through evolution by mechanisms such as unequal crossing over followed by random genetic drift or natural selection [1,2]. Recent studies indicate that the distribution of genes in genomes does not always happen at random [3-5]. In the human genome, housekeeping genes show a strong tendency to cluster together [6], and genes that participate in the same pathway also tend to lie adjacent to each other in the genome [5,7,8]. Moreover, several studies indicate that adjacent genes in human seem to co-express regardless of their intergenic distance [9-11]. Similar phenomena have been observed in *Drosophila*, nematode, and yeast [12-16]. Among these observations, the co-expression of adjacent pairs is crucial because changes in such genome organization could alter the co-regulated transcription over the pairs [11,12].

How co-expressed genes are regulated is still unclear. Two major mechanisms proposed are alterations of chromatin structure and sharing of the same regulatory elements [3,5,15]. The open conformation of the chromatin structure is required for genes to be transcribed into RNAs and thus become expressed. A general hypothesis is that clusters of genes in the same chromatin domain have a higher chance to be expressed simultaneously than genes located in different chromatin domains [5,17]. Alternatively, *cis*-regulatory elements could behave like fine modules that alter gene expression locally. Therefore, adjacent pairs with common upstream activation sites (UAS) or shared regulatory systems are more likely to be co-expressed [9,12].

Several attempts have been made to investigate the mechanism for co-expression of adjacent gene pairs. In human, the abundance of divergent pairs relative to convergent and tandem pairs has been reported [11], and the common CpG islands that were often found between divergent pairs were known to be associated with an "open" or "active" chromatin [11,18,19]. However, co-expressed groups of adjacent genes spanning 20–200 kilobases in the *Drosophila *genome did not show any correlation with known chromosomal structures [10,16]. Later, the idea of co-expression among clustered genes was rejected by Thygesen and Zwinderman [13], whose study also failed to discover any correlation between the chromatin domain and co-expressed genes in *Drosophila*.

It is evident that in yeast adjacent gene pairs display stronger co-expression than random pairs do [20]. Kruglyak and Tand [12] proposed that some co-expressed pairs resulted from sharing a single regulatory system, despite the fact that many genes controlled by separate regulatory systems may also have highly co-expressed patterns. Hurst *et al. *[9] also concluded that divergent orientation is dominant for co-regulation and for conservation of pairs, but the finding had weak statistical support. Although these studies suggested that the sharing of a common UAS plays an important role in regulating co-expressed pairs, and that divergent pairs are more likely to share the same regulatory system, the co-expression level (defined by correlation coefficient) of divergent pairs is not significantly higher than that of tandem pairs with a similar intergenic distance [20]. The relative contribution of the two major mechanisms to the co-expression of adjacent genes is still in debate for different organisms.

Recently, Byrnes *et al. *[21] proposed that the majority of gene loss in yeast happened after whole-genome duplication (WGD) by single-gene deletion. Their observation implied that adjacent gene pairs were not preserved after WGD. On the other hand, several studies indicated that adjacent pairs were conserved in some organisms due to the sharing of regulatory elements [4,22]. To investigate the contribution of regulatory elements to the co-expression of adjacent pairs, we first examined the conservation of adjacency in five yeast species. It is of particular interest to study the conservation of adjacent pairs using yeast species which have undergone WGD, because the duplicated adjacent relationship would in theory be free of evolutionary selection. Importantly, the advancement of technology has led to the establishment of databases of transcription factors (TF) and transcription factor binding sites (TFBSs). These tools allow researchers to investigate the mechanism for co-expression of adjacent pairs by studying sharing of common regulatory systems. Herein, we present a comprehensive examination of the intergenic regions between adjacent genes to inquire whether these pairs frequently share common TFs. Our study provides clear evidence that sharing of the common TFs is not an exclusive component of the driving force in co-regulation of adjacent gene pairs in yeast.

## Results

### Conservation of adjacent pairs in five yeast species

In order to investigate the conservation of adjacent pairs during the evolution of yeast, we collected *S. cerevisiae *orthologues from four *Saccharomyces sensu stricto *species (Table 1). In all cases, the proportions of conservation for different adjacent patterns (i.e. divergent pairs, convergent pairs and tandem pairs) were similar to those for random sampling composition (*p *> 0.05, chi-square test), suggesting that the three adjacent patterns have equal chances to be preserved. In more detailed analysis of stringently conserved pairs (definitions in Methods), we found that only a small fraction of adjacent pairs had maintained their particular adjacency relationships across these species. The conservation ratios are relatively low compared to those of the adjacent pairs found in *S. cerevisiae *(94/1491 for divergent pairs, 95/1474 for convergent pairs, and 156/2737 for tandem pairs) (Table 1). The low preservation ratios indicate that the adjacent relationship is not tightly maintained during the course of yeast evolution.

**Table 1:** Summary of the orthologous adjacent genes in *Saccharomyces sensu stricto *species relative to the 5,702 gene pairs in *S. cerevisiae*.

	Annotated ORFs^a ^	Orthologous genes	Orthologous adjacent pairs^b^	Divergent pairs^e^	Convergent pairs^e^	Tandem pairs^e^
*S. cerevisiae*	6310	5743	5702	1491	1474	2737
*S. castellii*	4681	3857	2053	528	604	921
*S. bayanus*	4970	4642	3975	1029	1051	1895
*S. kudriavzevii*	3778	3212	2376	637	647	1092
*S. mikatae*	3109	2435	1609	437	424	748
Stringently conserved pairs (ratio)^c^				94 (6.30%)	95 (6.45%)	156 (5.70%)
Loosely conserved pairs (ratio)^d^				942 (63.18%)	973 (66.01%)	1667 (60.91%)

^a^For analysis, the annotated ORFs exclude dubious and silent ORFS as well as overlapping genes.

^b^The orthologous adjacent pairs of the *sensu stricto *species are determined based on conservation of the orthologs as well as their adjacency relationship in *S. cerevisiae*.

^c^Gene pairs with adjacency relationship preserved in all five species for the orthologous pairs.

^d^Gene pairs with adjacency relationship preserved in any three species, or in *S. castelli *with at least one another yeast species used in this study.

^e^The proportions of conservation for different adjacent patterns are similar to those for random sampling composition (*p *> 0.05, chi-square test).

### Co-expression of conserved adjacent pairs

**Figure 1:**
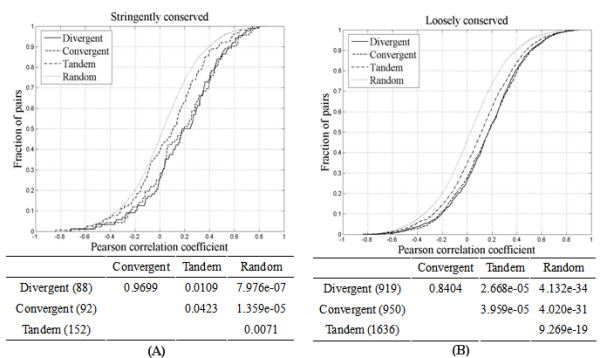
Comparison of co-expression level among three adjacent patterns. The upper figure illustrates the cumulative distribution of pairwise coefficients. The lower table indicates the significance suggested by KS test (*p *value). *A*, stringently conserved pairs, and *B*, loosely conserved pairs.

As shown in Fig. 1A, all three pairing types of stringently conserved pairs have significant levels of co-expression compared to that of random pairs (*p *< 0.0001, KS test). However, there is no obvious difference in co-expression levels between the three adjacent patterns (*p *> 0.01, KS test). In particular the divergent pairs did not show a higher co-expression as expected. On the contrary, in comparing the expression dataset of loosely conserved pairs, convergent and divergent pairs displayed higher co-expression levels than did tandem pairs with statistical support (*p *< 0.0001, KS test) (Fig. 1B).

**Figure 2:**
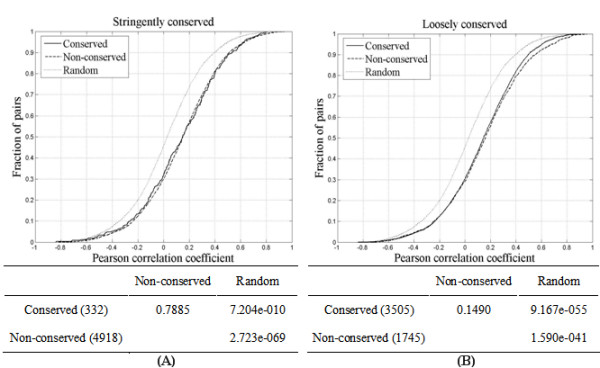
Comparison of co-expression level between conserved adjacent pairs and non-conserved adjacent pairs. *A*, stringently conserved pairs and *B*, loosely conserved pairs.

To investigate the conservation of co-expression tendency in adjacent pairs, the co-expression levels between conserved pairs and non-conserved pairs were compared. Expression patterns are available for 332 stringently and 3,505 loosely conserved adjacent pairs (Fig. 2). Neither dataset showed significant differences in co-expression level between conserved and non-conserved adjacent pairs (*p *> 0.01, KS test). Therefore, conservation of adjacency does not necessarily have an effect on the co-expression of adjacent genes.

### Commonly shared TFs of adjacent pairs

Since the co-expression level is not particularly high in conserved versus in non-conserved adjacent pairs, it was of great interest to address whether co-expression of adjacent pairs resulted from shared regulatory system [12]. In our analysis the proportion of adjacent pairs sharing common TFs was relatively low (Table 2). In addition, among the conserved adjacent pairs (including both stringently and loosely conserved), only about 8.1% of them shared single common TF and 4.4% shared multiple TFs (Table 2). The small fraction of sharing TFs among conserved adjacent pairs suggests that the co-expression of adjacent genes does not necessarily correlate with the shared regulatory system. Interestingly, the portion of sharing TFs among conserved adjacent pairs is similar to that found in non-conserved adjacent pairs, implying that adjacent pairs with shared TFs are not necessarily subjected to a stronger evolutionary constraint than those without shared TFs.

**Table 2:** The proportions of commonly shared TFs of conserved adjacent pairs and non-conserved pairs.

	No TF in common	Only one TF in common	Multiple TFs in common
Conserved (Stringently)	87.54%	8.11%	4.35%
Non-conserved (Stringently)	88.91%	7.26%	3.83%
Conserved (Loosely)	87.47%	8.26%	4.27%
Non-conserved (Loosely)	91.13%	5.71%	3.16%
Random^a^	98.34%	1.52%	0.14%

^a^5000 random pairs from *S. cerevisiae *were used for analysis.

**Figure 3:**
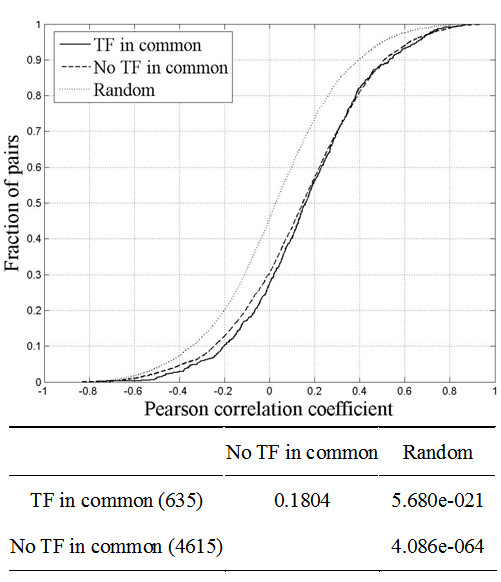
Comparison of co-expression level in adjacent pairs with shared TFs to those without shared TFs.

To explore whether adjacent pairs with shared TFs are more likely to be co-expressed, we compared the co-expression level of adjacent pairs with common TFs to those without. The result showed that both groups have significantly higher levels of co-expression relative to that of random pairs, but no significant difference was detected between the two groups (*p *> 0.01, KS test) (Fig. 3). This suggests that sharing of TFs may not be the main cause for the co-expression of adjacent pairs.

We acknowledge the potential bias and noise that microarray data may bear. To circumvent this problem, we also analyzed the condition-specific datasets separately (see Additional file 1 Fig. 1, 2, and 3) and obtained observations similar to those using the merged dataset.

### The co-expression of PAC- and RRPE-regulated divergent pairs

Since the proportion of adjacent pairs sharing TFs is low as aforementioned, it is important to inquire whether the shared TFs in divergent pairs are more likely to co-regulate the divergent genes. We present a case here to illustrate the effects of sharing regulatory system on co-expression. Beer and Tavazoie studied two computationally discovered sequence elements, PAC (polymerase A and C) and RRPE (rRNA processing element), which are considered to have combinatorial regulations on their target genes [23]. The authors found very similar expression patterns among genes with PAC located within 140 bp and RRPE within 240 bp of the ATG start codon, respectively.

**Figure 4:**
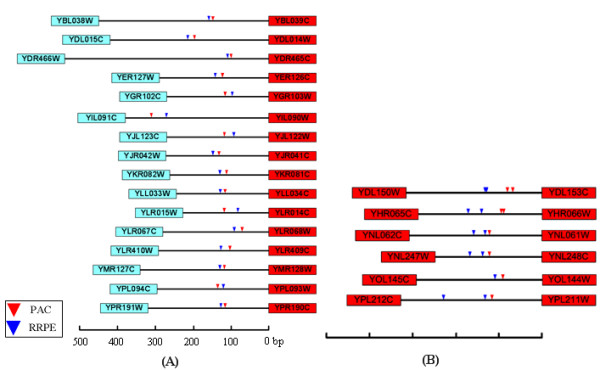
Locations of PAC and RRPE elements in 22 divergent gene pairs. *A*, sixteen divergent pairs with a single occurrence of the PAC-RRPE element; all the genes near the *cis*-elements are assigned to positive samples (red) except YIL090W-YIL091C. *B*, six gene pairs carry duplicate elements in their shared promoter region and are all considered positive samples for co-expression. Rectangles represent ORFs, lines between the ORFS are intergenic regions between the pair, and triangles indicate the binding sites for PAC and RRPE, respectively. Ruler at the bottom measures chromosomal distance.

To investigate whether such a pattern or any particular spatial arrangement of *cis*-regulatory motifs exists in divergent relationships, we selected 22 divergent pairs separated by an intergenic region of less than 400 bp and carrying both PAC and RRPE elements as annotated in the MYBS database [24]. Their expression profiles were extracted and compared using the method described above. In this analysis, sixteen divergent pairs revealed no sign of co-expression between the adjacent partners, while the other six pairs showed positive correlations for expression. Interestingly, the sixteen independently expressed pairs bore only a single occurrence of RRPE and PAC, in which the genes proximal to the motifs displayed similar expression patterns while the distal ones showed little correlation (Fig. 4A). In contrast, the remaining six divergent pairs exhibited co-expression of flanking genes on both sides, and they all carried multiple occurrences of RRPE or PAC in their common promoter regions (Fig. 4B).

Two implications can be drawn from this analysis. First, TFs tend to exert stronger regulatory effects on the gene proximal to their binding sites in a divergent pair. Second, sharing of TFs *per se *does not warrant co-regulation of adjacent genes, yet an increase in the motif occurrences may ensure simultaneous modulation on both sides in the situations where co-regulation is required. Altogether our results suggest that genes in a divergent pair do not necessarily use the same regulatory machinery, which in turn may lead to differential expression between the pair partners.

## Discussion

### Low conservation ratio of adjacent pairs in five yeast species

We compared the adjacency relationships of gene pairs among five yeast species which had undergone WGD and found random distribution for all three adjacent patterns. The evidence supported the hypothesis that the selection on types of adjacency along the *S. cerevisiae *lineage was neutral after WGD [21]. This neutrality also explained our observation of a low proportion of conserved adjacent pairs in five yeast species (e.g. 5.7~6.45% for the stringently conserved group). Similar results were found in orthologous gene pairs between the *S. cerevisiae *and *C. albicans *genomes which had diverged before WGD [9]. Since adjacent pairs have a tendency to co-express in yeast [20], observations from these studies contradict the hypothesis that adjacent pairs with co-expression patterns are more likely to maintain the adjacent relationship during evolution [4,11,22]. This implied that co-expression of adjacent pairs may be due to other mechanisms such as chromatin opening.

In contrast to yeast, a higher proportion of conserved adjacent pairs were observed in the genomes of mammalian lineage [11,22]. It is possible that the selection strength and/or mechanism over adjacency is different in yeast than in human [11,22]. It is also interesting to note that the ratios of conserved divergent, convergent, and tandem pairs are similar. This leads to the conclusion that for yeast divergent relationship is not appreciably favored by selection, even though these pairs are more likely to share a regulatory system and thus are more likely to display co-expression. Importantly, this notion is also different from that drawn from the vertebrate genomes, in which the conservation ratio of divergent pairs is higher than that of tandem pairs, suggesting a negative selection on the separation of divergent pairs during evolution of vertebrates [4].

It is proposed that the conservation of divergent pairs in human has functional importance. This hypothesis is supported by the significant expression correlation and functional association among divergent pairs [4,10,11,22]. Although several cases in yeast have shown functional associations for conserved divergent pairs [8,12], a higher co-expression level in divergent pairs could not be detected when compared to tandem pairs [20]. Consistent with this finding, we found no difference in the co-expression level among three adjacency patterns for the stringently conserved group, supporting the observation of neutrality in adjacency types. In addition, we found the co-expression levels of conserved adjacent pairs and non-conserved adjacent pairs to be approximately the same in yeast, indicating that the adjacent relationship of co-expressed pairs is free from selection constraint in yeast. It seems that a bias toward divergent gene organization is only observed in the lineage leading to mammals [22]. If this is true, a possible explanation is that the mechanisms concerning the co-expression of adjacent pairs in yeast are different from those in mammals. For example, mechanisms such as sharing of *cis*-regulatory elements and antisense transcription, both of which explained the co-expression of human adjacent genes [5,25], are actually rare in yeast genome [26,27].

### Low ratio of sharing common TFs in adjacent pairs

It has been suggested that when adjacent yeast genes are controlled by a single regulatory system, their expression patterns should be highly correlated [12,20]. In order to investigate whether shared TFs in adjacent pairs are responsible for the co-expression, we collected the TF information from adjacent pairs of *S. cerevisiae *for further analysis. Surprisingly, the ratio of adjacent pairs with shared TFs is low (about 12%). A similar trend is observed when the dataset is separated into conserved and non-conserved adjacent pairs, indicating that such feature is not particularly favored by selection. Therefore, it is reasonable to infer that co-expression of adjacent pairs in yeast does not merely result from sharing the TF-based regulatory system. This is also contrary to the findings that in human a high proportion of the adjacent pairs share a regulatory system which consequently drives co-expression of neighboring genes [11,22].

### A case study from the co-expression of PAC and RRPE

It is commonly believed that genes with the same regulators have similar expression profiles. However, we observed that the co-expression level of TF-sharing adjacent pairs is not higher than that of those without common TFs. We performed a case study on PAC and RRPE, two combinatorial *cis*-acting sequences whose target genes are expected to display high levels of co-expression. But our analysis showed the contrary that only 6 out of 22 divergent pairs had similar expression profile, and 72% (16 out of 22) of the divergent pairs are not co-expressed. Furthermore, the six co-expressed divergent pairs appear to have independent *cis*-regulatory elements. These results suggest that the shared regulatory system of adjacent genes in *S. cerevisiae *is not highly relevant to their co-expression.

Considering the low prevalence of sharing TFs and the lack of selection constraint on adjacency of adjacent pairs, one possible explanation for the co-expression phenomenon is chromatin modifications [3,15,29]. Mechanisms such as histone acetylation, deacetylation and DNA methylation, may contribute significantly to the co-expression of neighboring genes in *S. cerevisiae *[25,28]. Detailed analyses of transcription pattern as well as chromatin structure of co-expressed genes are required to shed light on the questions raised by this report.

## Conclusion

The purpose of this study arose from speculating on the impact that sharing of TFs might have on driving concurrent expression of adjacent gene pairs. We found that gene adjacency was not strongly favored during yeast evolution. Furthermore, the analysis on co-expression in adjacent gene pairs and shared TFs showed an indistinct relationship. Albeit the bias or noise potentially present in microarray data, the clear result of the divergent pairs co-regulated by PAC and RRPE led us to conclude that the shared TFs can not fully explain the co-expression of divergent pairs.

In summary, our study does not refute the contribution of commonly shared TFs to co-regulation of adjacent genes in yeast, but our finding does suggest that TF sharing is not the sole determinant of such regulation. We believe that during evolution yeasts have developed a sophisticated regulatory system which integrates both TF-based and non-TF based mechanisms(s), of which the latter may account for a greater extent in driving co-expression of neighboring genes. This integrative regulatory system allows yeasts to simultaneously modulate expression of neighboring genes in order to adapt to changing environments rapidly and efficiently.

## Methods

### Identification of orthologous adjacent pairs in five yeast species

The genome sequences and annotations of five yeast species (including *Saccharomyces cerevisiae, Saccharomyces castellii, Saccharomyces bayanus*, *Saccharomyces kudriavzevvi *and *Saccharomyces mikatae*) were downloaded from Saccharomyces Genome Database (SGD, ). There were 6310, 4681, 4970, 3778, and 3109 annotated ORFs from these genomes, respectively. Gene pairs in *S. cerevisiae *were identified by their relative position in the genome. Dubious and silent ORFs were excluded from analysis. Overlapping genes were also removed because they might have biased the expression analysis. Finally a total of 5743 genes of *S. cerevisiae *were used for analysis. Based on the positional annotation in SGD they were categorized into groups of divergent pairs, convergent pairs and tandem pairs, adding up to 5702 adjacent pairs detected in *S. cerevisiae*.

Using *S. cerevisiae *as the reference genome, orthologous ORFs were identified in *S. castellii *(3857), *S. bayanus *(4642), *S. kudriavzevvi *(3212), and *S. mikatae *(2435) (Table 1). Except for *S. cerevisiae*, we determined adjacent pairs for the remaining four yeast species by mapping all the ORFs to their contigs according to the annotated sequences. The ORFs within the same contigs were sorted based on their hit positions. Adjacent relationship was then designated by their relative positions and orientations annotated in SGD. We identified 2053, 3975, 2376, and 1609 orthologous adjacent pairs in *S. castellii*, *S. bayanus*, *S. kudriavzevvi*, and *S. mikatae*, respectively. (Table 1)

We considered an adjacent pair **conserved **if the neighboring ORFs were orthologues of adjacent genes in *S. cerevisiae *and meanwhile retained the same orientation pattern. If one (or both) genes of an adjacent pair in *S. cerevisiae *were missing or the pairing orientation was different in other species, the pair was ascribed to the non-conserved group. The conserved pairs were then classified into stringently conserved and loosely conserved groups according to their degree of conservation. An adjacent pair was considered stringently conserved if the adjacent relationship was preserved in all five yeast species. The loosely conserved group refers to the pairs that have an adjacent relationship preserved in any three of the five yeast species, or preserved in *S. castellii *and one another of the four yeast species. This is because that *S. castellii *is the most distantly related species among the five, and the chance of convergent evolution is remote. As a result, there were 345 stringently conserved pairs and 3,582 loosely conserved pairs. Among those, there were 94, 95, and 156 stringently conserved pairs and 942, 973, and 1,667 loosely conserved pairs for the divergent, convergent, and tandem categories, respectively.

To compare the preserved patterns among these five yeast species, the relative ratios of three adjacent patterns were analyzed by chi-square test using a random sampling as reference (0.25 : 0.25 : 0.5) (Table 1).

### Evaluation of the expression correlation between conserved adjacent pairs

We selected four *S. cerevisiae *microarray datasets for the expression analysis, including *alpha *[30], *cdc *[30], *crz1p *[31], and *env *[32]. Both the *alpha *and *cdc *datasets are time course expression profiles encompassing two to three cell cycles after release from growth arrest. The *alpha *data were obtained from cells treated with *alpha*-factor transiently, and the *cdc *data was collected from a *cdc15-2 *temperature sensitive mutant which resumed growth after release from heat shock. For the *crz1p *dataset, yeast cells were triggered for ionic signaling by either calcium (Ca^2+^) or sodium (Na^+^). The *env *dataset contains expression profiles of yeast cells exposed to diverse environmental perturbations. Each array was normalized so that the log ratios had a mean of zero. To avoid potential discrepancy between arrays due to factors specific to each condition, we merged these four array data into one large dataset and used the *Pearson *coefficient to calculate the co-expression level for each adjacent pair.

To investigate whether conserved adjacent pairs had a higher tendency to be co-expressed, we compared for the conserved group the expression correlations of divergent pairs, convergent pairs, and tandem pairs to those of a group of 5,000 non-adjacent random pairs. We used the Kolmogorov-Smirnov (KS) test to examine whether two groups of gene pairs were co-expressed to different extents. The KS test is a nonparametric test which determines if two distributions differ significantly. The KS test calculates the maximum vertical deviation (*D*) between the empirical distribution functions of the two groups to determine whether the two datasets are drawn from the same distribution. Let *x *be the expression correlation of an ORF pair over all experimental points. Let *f*_*i*_(*x*) be the density function of *x *for the gene pairs in group *i*, and *F*_*i*_(*x*) be the function of corresponding cumulative distribution. For groups *i *and *j*, if the statistic *D *is significantly large, we infer that the two groups of gene pairs are from two distinct distributions and are expressed differentially. Similarly, we used the KS test to examine the significance of the differences between conserved adjacent pairs and non-conserved adjacent pairs.

### Identification of transcription factors shared among adjacent pairs

To understand whether co-expression of adjacent pairs is mainly due to sharing of the same regulatory system, we studied the correlation of co-expression level to the presence and the number of commonly shared TFs. We collected the TF information from MYBS [24], a web-based service that identifies TFBSs with comprehensive annotation. MYBS integrates an array of predicted and known transcription factor binding sites (TFBSs) with a calculated position weight matrix (PWMs) and incorporates DNA-binding affinity data from chromatin-immunoprecipitation microarray experiments (ChIP-chip) as well as the phylogenetic footprinting data of TFBSs from eight related yeast species.

In this study, we considered a TF to regulate a gene if: 1) its binding to the gene was supported by a *p*-value less than 0.01 in the ChIP-chip experiment; 2) there existed a short sequence pattern satisfying the PWM's threshold; and 3) the sequence pattern was conserved in at least one of the four *Saccharomyces sensu stricto *species.

For more detailed comparison, we classified the adjacent pairs into three groups of pairs without shared TFs, pairs with one TF in common, and pairs with multiple TFs in common. Again, we used the KS test to examine whether shared TFs were relevant to the co-expression level between the pair.

## Competing interests

The author(s) declares that there are no competing interests.

## Authors' contributions

HKT and DW formulated the studies, participated in the experimental design, and drafted the manuscript. PCS and CHS participated in the program design of the study and performed the statistical analysis. MYJL commented on the study and contributed in drafting the manuscript. All authors read and approved the final manuscript.

## Supplementary Material

Additional file 1 Comparison of expression level per condition-specific dataset. In order to show the consistency of each dataset, the data provided represent the statistical analysis of expression level using condition-specific dataset separately. Click here for file

## References

[B1] Marais G, Charlesworth B (2003). Genome evolution: recombination speeds up adaptive evolution. Curr Biol.

[B2] Marais G, Mouchiroud D, Duret L (2001). Does recombination improve selection on codon usage? Lessons from nematode and fly complete genomes. Proc Natl Acad Sci U S A.

[B3] Semon M, Duret L (2006). Evolutionary origin and maintenance of coexpressed gene clusters in mammals. Mol Biol Evol.

[B4] Li YY, Yu H, Guo ZM, Guo TQ, Tu K, Li YX (2006). Systematic analysis of head-to-head gene organization: evolutionary conservation and potential biological relevance. PLoS Comput Biol.

[B5] Hurst LD, Pal C, Lercher MJ (2004). The evolutionary dynamics of eukaryotic gene order. Nat Rev Genet.

[B6] Lercher MJ, Urrutia AO, Hurst LD (2002). Clustering of housekeeping genes provides a unified model of gene order in the human genome. Nat Genet.

[B7] Lee JM, Sonnhammer EL (2003). Genomic gene clustering analysis of pathways in eukaryotes. Genome Res.

[B8] Zhang X, Smith TF (1998). Yeast "operons". Microb Comp Genomics.

[B9] Hurst LD, Williams EJ, Pal C (2002). Natural selection promotes the conservation of linkage of co-expressed genes. Trends Genet.

[B10] Takai D, Jones PA (2004). Origins of bidirectional promoters: computational analyses of intergenic distance in the human genome. Mol Biol Evol.

[B11] Trinklein ND, Aldred SF, Hartman SJ, Schroeder DI, Otillar RP, Myers RM (2004). An abundance of bidirectional promoters in the human genome. Genome Res.

[B12] Kruglyak S, Tang H (2000). Regulation of adjacent yeast genes. Trends Genet.

[B13] Thygesen HH, Zwinderman AH (2005). Modelling the correlation between the activities of adjacent genes in Drosophila. BMC Bioinformatics.

[B14] Blumenthal T, Evans D, Link CD, Guffanti A, Lawson D, Thierry-Mieg J, Thierry-Mieg D, Chiu WL, Duke K, Kiraly M, Kim SK (2002). A global analysis of Caenorhabditis elegans operons. Nature.

[B15] Fukuoka Y, Inaoka H, Kohane IS (2004). Inter-species differences of co-expression of neighboring genes in eukaryotic genomes. BMC Genomics.

[B16] Spellman PT, Rubin GM (2002). Evidence for large domains of similarly expressed genes in the Drosophila genome. J Biol.

[B17] Sproul D, Gilbert N, Bickmore WA (2005). The role of chromatin structure in regulating the expression of clustered genes. Nat Rev Genet.

[B18] Antequera F (2003). Structure, function and evolution of CpG island promoters. Cell Mol Life Sci.

[B19] Adachi N, Lieber MR (2002). Bidirectional gene organization: a common architectural feature of the human genome. Cell.

[B20] Cohen BA, Mitra RD, Hughes JD, Church GM (2000). A computational analysis of whole-genome expression data reveals chromosomal domains of gene expression. Nat Genet.

[B21] Byrnes JK, Morris GP, Li WH (2006). Reorganization of adjacent gene relationships in yeast genomes by whole-genome duplication and gene deletion. Mol Biol Evol.

[B22] Koyanagi KO, Hagiwara M, Itoh T, Gojobori T, Imanishi T (2005). Comparative genomics of bidirectional gene pairs and its implications for the evolution of a transcriptional regulation system. Gene.

[B23] Beer MA, Tavazoie S (2004). Predicting gene expression from sequence. Cell.

[B24] Tsai HK, Chou MY, Shih CH, Huang GT, Chang TH, Li WH (2007). MYBS: A comprehensive web server for mining transcription factor binding sites in yeast. Nucleic Acids Res.

[B25] Ge X, Wu Q, Jung YC, Chen J, Wang SM (2006). A large quantity of novel human antisense transcripts detected by LongSAGE. Bioinformatics.

[B26] David L, Huber W, Granovskaia M, Toedling J, Palm CJ, Bofkin L, Jones T, Davis RW, Steinmetz LM (2006). A high-resolution map of transcription in the yeast genome. Proc Natl Acad Sci U S A.

[B27] Miura F, Kawaguchi N, Sese J, Toyoda A, Hattori M, Morishita S, Ito T (2006). A large-scale full-length cDNA analysis to explore the budding yeast transcriptome. Proc Natl Acad Sci U S A.

[B28] Kalmykova AI, Nurminsky DI, Ryzhov DV, Shevelyov YY (2005). Regulated chromatin domain comprising cluster of co-expressed genes in Drosophila melanogaster. Nucleic Acids Res.

[B29] Emre NC, Berger SL (2006). Histone post-translational modifications regulate transcription and silent chromatin in Saccharomyces cerevisiae. Ernst Schering Res Found Workshop.

[B30] Spellman PT, Sherlock G, Zhang MQ, Iyer VR, Anders K, Eisen MB, Brown PO, Botstein D, Futcher B (1998). Comprehensive identification of cell cycle-regulated genes of the yeast Saccharomyces cerevisiae by microarray hybridization. Mol Biol Cell.

[B31] Yoshimoto H, Saltsman K, Gasch AP, Li HX, Ogawa N, Botstein D, Brown PO, Cyert MS (2002). Genome-wide analysis of gene expression regulated by the calcineurin/Crz1p signaling pathway in Saccharomyces cerevisiae. J Biol Chem.

[B32] Gasch AP, Spellman PT, Kao CM, Carmel-Harel O, Eisen MB, Storz G, Botstein D, Brown PO (2000). Genomic expression programs in the response of yeast cells to environmental changes. Mol Biol Cell.

