# Supplementary material for: Co-expression of adjacent genes in yeast cannot be simply attributed to shared regulatory system
Source: BMC Genomics. 2007 Oct 3;8:352. doi: 10.1186/1471-2164-8-352 (PMC2045684; doi:10.1186/1471-2164-8-352)
Supplement: Additional file 1 — Comparison of expression level per condition-specific dataset. In order to show the consistency of each dataset, the data provided represent the statistical analysis of expression level using condition-specific dataset separately. [file 1471-2164-8-352-S1.pdf]

Figure Legends

S-Fig. 1

Comparison of co-expression level for three adjacent patterns per condition-specific dataset (A: *alpha*, B: *cdc*, C: *crz1p*, D: *env*). The upper figure illustrates the cumulative distribution of pairwise coefficients. The lower table indicates the significance suggested by KS test (*p* value).

S-Fig. 2

Comparison of co-expression level between conserved adjacent pairs and non-conserved adjacent pairs. A: *alpha*, B: *cdc*, C: *crz1p*, D: *env*.

S-Fig. 3

Comparison of co-expression level between the adjacent pairs with shared TFs and those without shared TFs. A: *alpha*, B: *cdc*, C: *crz1p*, D: *env*.

S-Fig 1A

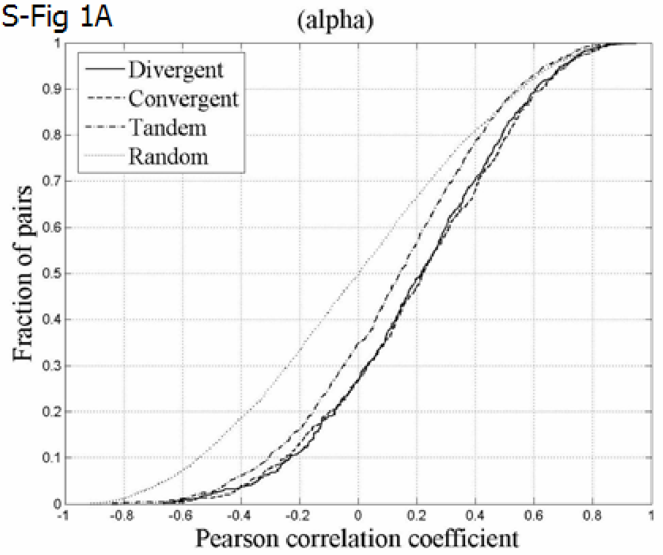

	Convergent	Tandem	Random
Divergent (594)	0.8941	0.0022	8.144e-028
Convergent (641)		9.948e-005	1.114e-028
Tandem (1020)			2.209e-024

S-Fig 1B

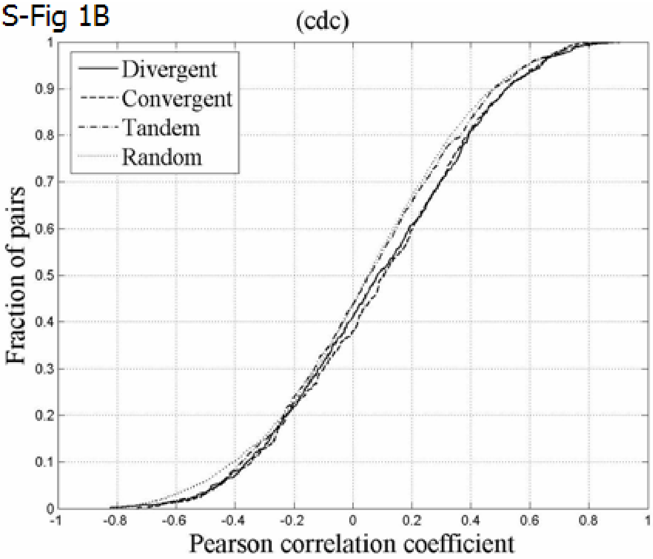

	Convergent	Tandem	Random
Divergent (707)	0.6730	0.0545	0.0023
Convergent (735)		0.0082	0.0002
Tandem (1264)			0.3337

S-Fig 1C

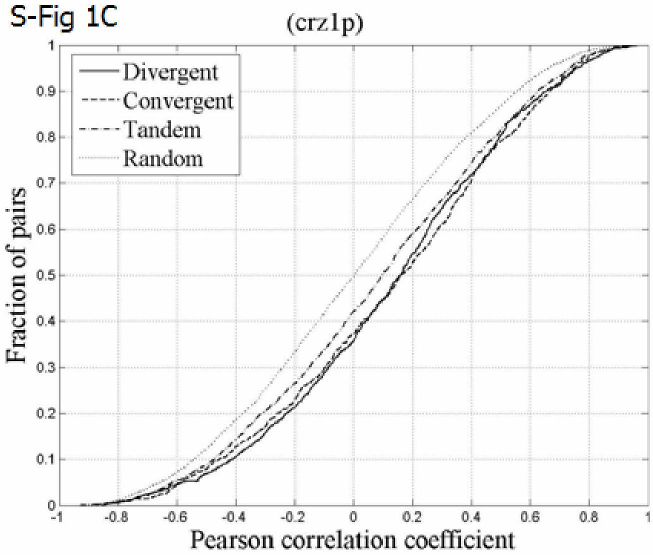

	Convergent	Tandem	Random
Divergent (903)	0.3529	0.0136	5.305e-014
Convergent (943)		0.0069	4.096e-015
Tandem (1613)			1.490e-008

S-Fig 1D

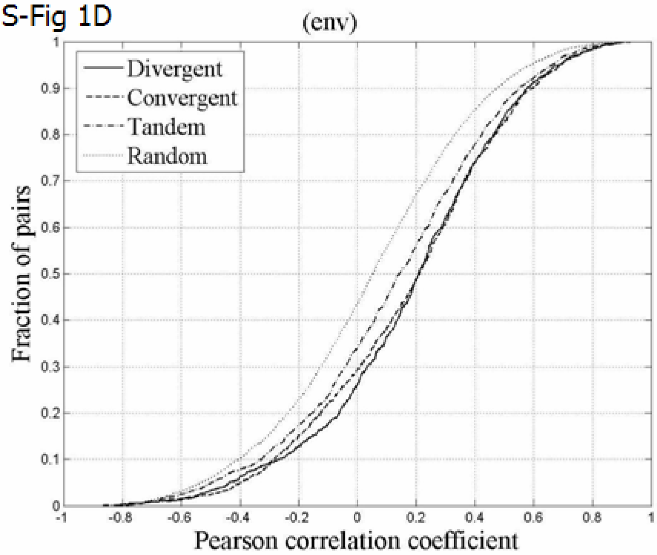

	Convergent	Tandem	Random
Divergent (917)	0.1686	3.756e-005	3.303e-028
Convergent (946)		0.0013	8.302e-025
Tandem (1628)			7.183e-015

S-Fig 2A

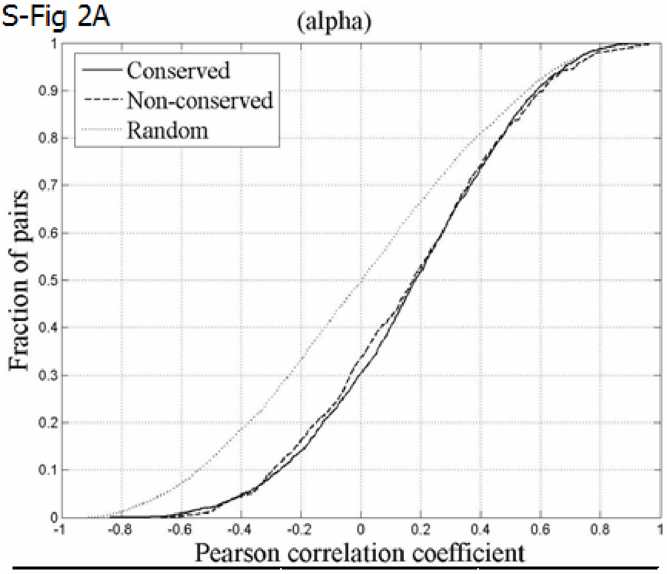

	Non-conserved	Random
Conserved (2255)	0.1462	9.883e-058
Non-conserved (1002)		9.592e-028

S-Fig 2B

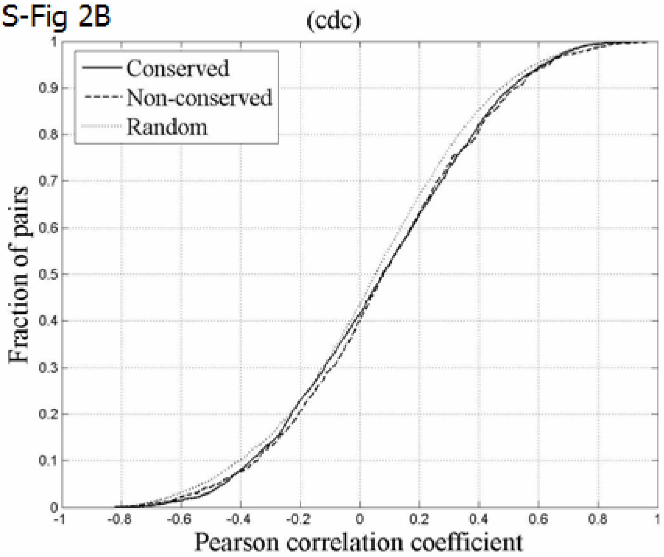

	Non-conserved	Random
Conserved (2706)	0.3913	0.0007
Non-conserved (1312)		0.0068

S-Fig 2C

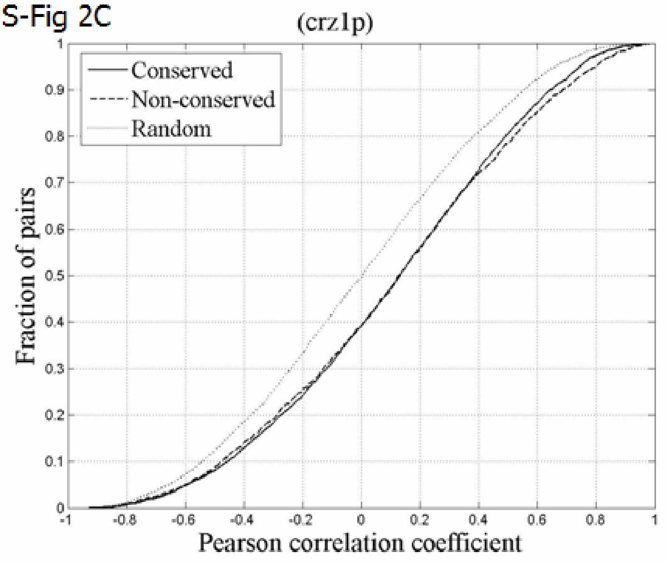

	Non-conserved	Random
Conserved (3450)	0.3841	2.488e-024
Non-conserved (1716)		2.519e-015

S-Fig 2D

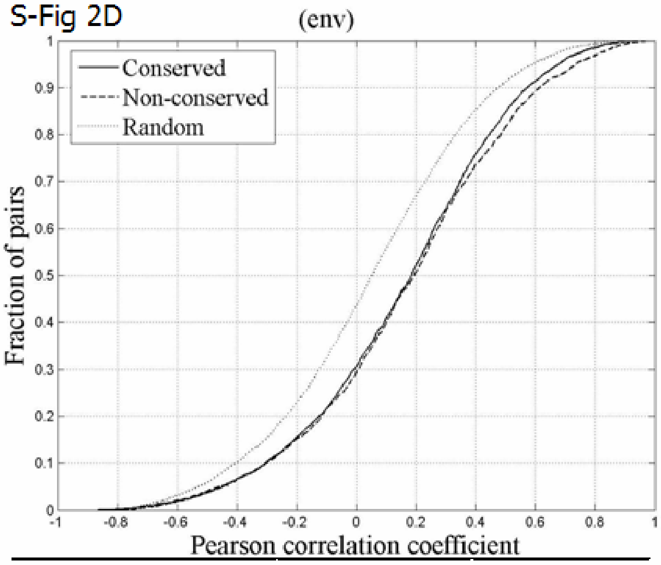

	Non-conserved	Random
Conserved (3491)	0.1265	4.905e-044
Non-conserved (1740)		1.407e-031

S-Fig 3A

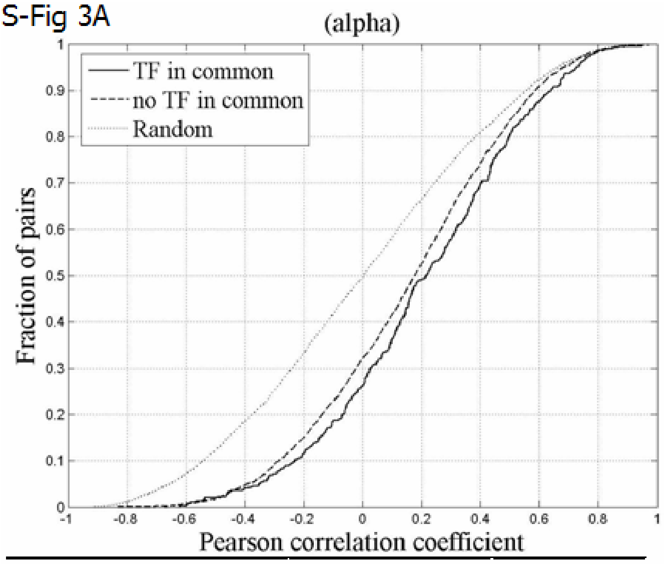

	No TF in common	Random
TF in common (390)	0.0569	2.463e-020
No TF in common (2867)		3.689e-061

S-Fig 3B

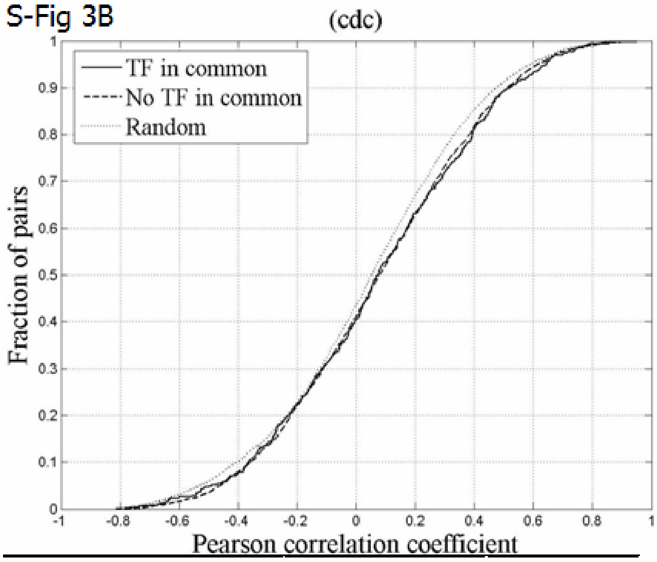

	No TF in common	Random
TF in common (495)	0.9702	0.0545
No TF in common (3523)		0.0006

S-Fig 3C

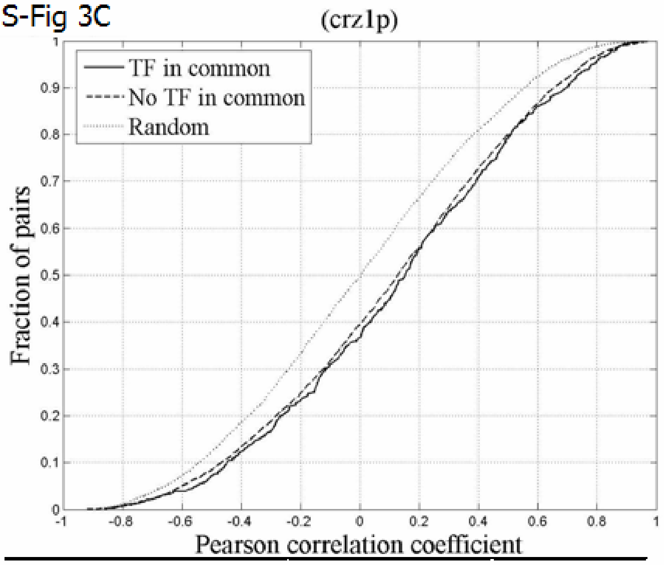

	No TF in common	Random
TF in common (622)	0.7165	2.462e-009
No TF in common (4544)		2.798e-026

S-Fig 3D

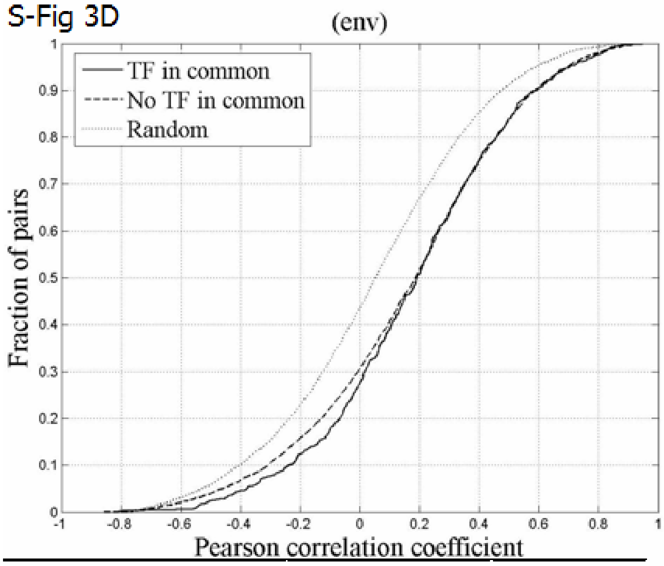

	No TF in common	Random
TF in common (632)	0.0958	2.926e-015
No TF in common (4599)		5.788e-052
